# Positive Impact of a Specialized Summer Camp on the Correlation Between Improved Mental Health and Glycemic Control in Pediatric Type 1 Diabetic Patients

**DOI:** 10.1155/pedi/4811222

**Published:** 2025-03-08

**Authors:** Lauren McManus, Colby Vinson, Dharak Patel, Casey Faichtinger, Zakariya Yazdani, Rikki Ray, Rhadika Patel, Matthew Stokell, Brooke Birks, Lesley A. Gardiner, Petra Rocic

**Affiliations:** Departments of Primary Care and Clinical Medicine and Physiology and Pharmacology, College of Osteopathic Medicine, Sam Houston State University, Conroe 77304, Texas, USA

**Keywords:** glycemic control, mental health, pediatric, psychosocial wellness, type I diabetes

## Abstract

Type 1 diabetes mellitus (T1DM) is associated with an increased risk of mental illness. In recent years, specialized summer camps for children and adolescents with type 1 diabetes have emerged, aimed at normalizing life with diabetes and building skills needed for optimal management of the condition. This project analyzed the effects of one such camp, Camp Sweeney, on glycemic control, physical health, and psychosocial wellbeing of camp attendees (children 5–17, mean age 14.4 years old) and their parents/caregivers. The standard Pediatric Quality of Life Inventory (PedsQL) was modified by the addition of questions pertaining to self-assessment of diabetes management, and questionnaires were distributed to parents and campers to complete at the start of and 2 months after completion of the camp. A total of 14 completed surveys (7 child/camper–parent/caregiver pairs) were collected and analyzed. Self-reported glycemic control (DM management), perceived overall quality of life (wellness), physical wellness, and psychosocial wellness improved after attendance of Camp Sweeney as reported by both campers/children (*Δ*17.86% DM management, *Δ*10.96% overall wellness, *Δ*16.25% psychosocial wellness) and their parents/caregivers (*Δ*16.07% DM management, *Δ*14.54% overall wellness, *Δ*17.86% psychosocial wellness). Importantly, we established a significant positive correlation between glycemic control (DM management) and overall wellness, psychosocial wellness, and average quality of life (correlation coefficient = 0.92, 0.80, and 0.94, respectively). While previous studies do provide some evidence that these types of camps improve the mental wellbeing of participants, this is the first study to establish a direct correlation between improved mental and psychosocial wellbeing and diabetes management.

## 1. Introduction

Type 1 diabetes mellitus (T1DM) can adversely affect the quality of life for children with the diagnosis and their caretakers [1]. If suboptimally managed, it is correlated with an increased risk of mental illness and decreased life expectancy [2]. One study has shown that the added stress on parents of children with T1DM increases the risk for long-term complications of diabetes and has adverse impacts on their children's quality of life [3]. The relentless demands of diabetes management can pose significant challenges, particularly for young patients and their families. Moreover, psychosocial factors such as diabetes-related distress, anxiety, depression, and low self-esteem are prevalent among individuals with T1DM and can negatively impact their overall well-being and glycemic control. A stronger correlation was observed between the severity of depression and acute symptoms of diabetes (hyper and hypoglycemia) than between glycemic control and diabetic complications [4].

Specialized summer camps for children and adolescents with T1DM have emerged as an effective intervention to address the unique psychosocial and educational needs of this population. These camps provide a supportive environment where participants can learn diabetes self-management skills, connect with peers facing similar challenges, and receive guidance from healthcare professionals specialized in diabetes care. By immersing participants in an environment focused on diabetes management, specialized summer camps aim to empower young individuals to take control of their health and improve their overall quality of life. The American Diabetes Association states that an important goal of these camps is to “enable children with diabetes to meet and share their experiences with one another while they learn to be more responsible for their condition” [5].

Accordingly, an accurate assessment of the impact of diabetes camps on psychological distress in children and adolescents with T1DM seems critically important. However, only a handful of recent studies have investigated the association between attending a diabetes camp and psychosocial outcomes, often with varying and often apparently contrasting outcomes, which cannot be easily explained [6–10]. Furthermore, only two of these studies also assessed the effect of camp attendance on glycemic control [7], and none of the studies examined the correlation between the camps' effects on psychosocial parameters and glycemic control.

The purpose of this study is to determine the effect of a specialized diabetes summer camp, Camp Sweeney, on the glycemic control and mental health of children and adolescents with T1DM. We will also evaluate whether there is a correlation between mental health and glycemic control in the camp attendees and whether the correlation is affected by camp attendance.

## 2. Materials and Methods

### 2.1. Study Location

The study was conducted at Camp Sweeney located in Callisburg, Texas. Camp Sweeney is a specialized summer camp for children and adolescents who have been diagnosed with T1DM. The camp focuses on normalizing optimal glycemic control and integrating type I diabetes care into normal daily life by providing knowledge and practical experience and building a life-long peer support network [9, 11]. Campers attend an 18-day camp session and follow a highly structured daily schedule [9, 11]. Throughout this time, their diabetes is intensively managed by medical staff through tight glycemic control, nutrition, and physical activities, and trained camp counselors facilitate complementary physical and psychosocial activities. Improved HbA1c values have been reported following Camp Sweeney sessions in the 2008, 2013, and 2018 cohorts [9, 11]. Additionally, participation in “PFC Life,” a group available to all Camp Sweeney attendees after completion of their session, provides life-long peer support for the campers [12].

### 2.2. Study Participants

The study employed a prospective longitudinal cohort design to investigate a correlation between attendance at Camp Sweeney, type 1 diabetes management, and descriptive markers of physical and mental health in a cohort of camp attendees who met the following criteria. The study was designed to collect information exclusively regarding the children/campers from the perspective of both the children and their parents/caregivers. We did not collect information regarding parent/caregiver wellness.

### 2.3. Inclusion Criteria

Children between 5 and 17 years of age diagnosed with T1DM participating in an 18-day session at Camp Sweeney during the summer of 2023 and their parents/caregivers. A total of 100 families were contacted, and 14 individuals, 7 child–parent pairs, who responded to both the pre- and post-camp surveys, were included in the study. Participant demographics are shown in Table 1.

### 2.4. Exclusion Criteria

Individuals younger than 5 or older than 17, those without T1DM, those absent from the program for longer than 3 days due to extenuating circumstances, those who failed to submit or complete the consent form, the pre- or the post-camp survey, or those who do not speak English.

### 2.5. Data Collection

The standard Pediatric Quality of Life Inventory (PedsQL) [13] was modified by the addition of two questions pertaining to self-assessment of glycemic control (child and parent/caregiver were asked to report HbA1c and/or repeated glucose measurements. The two added questions were as follows:

1. Camper glycemic control self-assessment (please use recent HbA1c or blood glucose measurements for assessment):
  a. Needs improvement.
  b. Good.
  c. Very good.
  d. Excellent.
2. How confident are you in your ability to manage your diabetes on your own (child)? How confident are in your child's ability to manage their diabetes on their own (parent)?
  a. Needs improvement.
  b. Good.
  c. Very good.
  d. Excellent.

Four survey questionnaires were constructed: (1) pre-camp child survey, to be completed by the child Camp Sweeney participant before the start of their 18-day camp session, (2) post-camp child survey, to be completed by the child Camp Sweeney participant 2 months after completion of their camp session, (3) pre-camp parent/caregiver survey, to be completed by a parent/caregiver of a Camp Sweeney participant before start of the 18-day camp session, and (4) post-camp parent/caregiver survey, to be completed by the parent/caregiver of a Camp Sweeney participant 2 months after completion of the camp session.

Informed consent was provided for all participants, and the study adhered to HIPAA regulations and was approved by the Sam Houston State University Institutional Review Board.

### 2.6. Data Analysis

**Figure 1:**
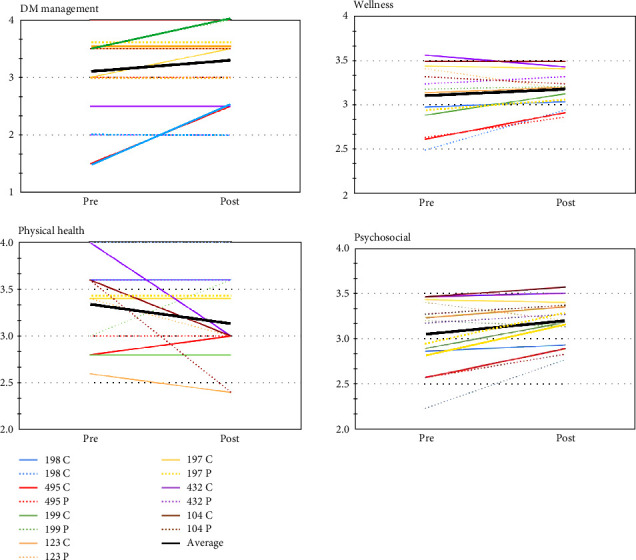
The individual results depicted here correspond with cumulative data shown in Table 2. Results from each individual child–parent pair (*n* = 7 pairs; *n* = 14 individuals) are shown pre- and post-attendance at Camp Sweeney for each of the four domains. Children (C) are depicted in solid lines and their parents (P) in dotted lines of the same color (numbers are randomly generated study identifiers used to track child–parent pairs after deidentification). Averaged results of all child and parent data are represented by the black lines in each of the four domains.

Surveys were deidentified, but pre-camp and post-camp surveys of each child and their parent/caregiver were tracked together. Questions (32 total) were grouped into three standard PedsQL domains: overall wellness (12 questions), physical wellness (4 questions), and psychosocial wellness (16 questions), and the diabetes management domain (DM, 2 questions) was added. Likert scale scores (0—never; 1—seldom; 2—sometimes; 3—often; 4—all the time) were converted into numerical values (0 = 0; 1 = 0.25 [25%]; 2 = 0.50 [50%]; 3 = 0.75 [75%]; 4 = 1.00 [100%]), and numerical values of individual questions were averaged for each domain. For DM management, the Likert scale (0—none; 1—needs improvement; 2—good; 3—very good; 4—excellent) was converted into numerical values as for the standard PedsQL domains. Individual participant scores are depicted in Figure 1.

The data from campers and their caregivers were first analyzed as individual data points (not averaged scores shown in Table 2) were by repeated, two-way ANOVA followed by the Bonferroni post hoc analysis. The differences between pre-camp and post-camp results were then calculated separately for campers (children) and their caregivers (parents) by averaging their scores in each domain pre-camp and post-camp attendance, then calculating the % difference between the pre- and post-camp averages for the campers (Child *Δ*) and their caregivers (Parent *Δ*).

Linear correlation analysis was performed on the difference of combined child and parent data (*Δ* Child and *Δ* Parent) for all domains. The Pearson correlation coefficients (*R*^2^) for those correlations are reported in Table 3, and highly significant positive correlations (*⁣*^*∗*^*p* < 0.05) are marked.

## 3. Results

A total of seven completed pre-camp and post-camp survey pairs (child/camper–parent/caregiver) were collected and analyzed. Self-reported glycemic control (DM management), perceived overall quality of life (wellness), and psychosocial wellness improved after attendance of Camp Sweeney as reported by both campers/children (*Δ*17.86% DM management, *Δ*10.96% overall wellness, *Δ*16.25% psychosocial wellness) and their parents/caregivers (*Δ*16.07% DM management, *Δ*14.54% overall wellness, *Δ*17.86% psychosocial wellness) (Table 2). There is also a trend toward the caregivers' perception of their children's quality of life, in the three standard PedsQL domains, to be higher than the children's. The cumulative data did not achieve statistically significant differences between groups pre- and post-camp. However, variability between individual study participants within groups was significant (two-way ANOVA for repeated measurements). Figure 1 illustrates individual pre- and post-camp results for all four domains.

The major novel aspect of this study is the correlation between the changes in diabetes management (DM management), physical and psychosocial wellness, which was highly significant (*R*^2^ = 0.9, 0.8, and 0.9 DM management vs. overall wellness, psychosocial wellness and average quality of life [average of all three PedsQL domains] (Table 3)), which suggest that the camp attendance and the difference (*Δ*) is important.

## 4. Discussion

In agreement with several similar studies, while positive trends were observed, our results did not achieve statistically significant differences in either DM management or any parameter of wellness before versus after attendance at Camp Sweeney (Figure 1 and Table 2). The small sample size is, of course, one possible reason for this outcome. In another study, very similar to our own in terms of parameters measured, the timeline of data collection, and with a small sample size (*n* = 20 child–parent participant pairs), camp attendance likewise showed no beneficial effects on glycemic control and no significant improvements in the psychosocial component [14]. Studies with much larger numbers of participants tend to show statistically significant improvements in indicators of overall wellness [6–8].

However, sample size alone does not account for differences in outcome. A systematic review and meta-analysis conducted by Hasan et al. [10], encompassing more that 500 participants in total, showed that significant evidence supported the benefits of diabetes camps on HbA1c values but that changes in quality of life showed only improvement trends without statistical significance. Vice versa, Gupta et al. [9] conducted a similar study at Camp Sweeney in 2016, with a sample size comparable to ours (*n* = 18 child–parent pairs), and showed a significant improvement in some psychosocial wellness indexes [9].

We think that the observed variability in outcomes between studies may be associated with variability in exact parameters measured in different studies. Studies typically report evaluation of overall umbrella categories, such as “wellness,” “psychosocial wellness,” “diabetes management,” or “stress.” However, detailed examinations of methods reveal that quite different components under these broad categories are actually addressed. For example, our current study uses the PedsQL to assess psychosocial wellness and finds no statistically significant improvement in this domain after attendance at Camp Sweeney. The 2016 study also uses the PedsQL and similarly finds no significant improvement in psychosocial wellness when using this test. Gupta et al. [9] also use a battery of other tests and find significant improvement in the psychosocial domain using the diabetes-specific QL and Pediatric Inventory for Parent (PIP) assessment. Similar situations arise with sub-categorization within categories. Stress, for example, has been used to describe assessments of stress related specifically to the accuracy of glucose level control/hypoglycemia [9], stress related to the future (health, work, etc.), stress related to social interactions because of diabetes, stress unrelated to diabetes in diabetic patients, etc. [10] The “social” component of psychosocial wellness can encompass: social anxiety, “social support-seeking as a patient pain-coping strategy,” level of participation in “normal” but optional social activities, or enjoyment/tolerance of required social activities (ex. school), etc. [9, 10, 14].

Thus, the apparent high degree of variable outcomes may, in fact, be due in part to the assessment of vastly different parameters. While detailed information is (in most cases) clearly presented in the publications, a cursory review, which is regrettably common today, finds only “unexplainable contradictions,” which can have disastrous consequences on interventions already considered nonessential. Perhaps well-intentioned research in an attempt to be comprehensive while simultaneously avoiding repetition has now led to a certain level of confusion, and the field might benefit from arriving at a consensus regarding a standardized evaluation matrix that could be applied to these types of studies to enable consistent assessment and better comparison of results between studies.

The effectiveness of diabetes camps on mental health may also be significantly influenced by the use of insulin pumps among the camp participants. The use of insulin pumps became common in children and adults in the early 2000s. Several primary studies and meta-analyses demonstrated highly significant correlations between insulin pump use and improved quality of life for diabetic patients [15]. All but one of the participants in our study used insulin pumps. Thus, it is conceivable that the lack of significant increase in the psychosocial wellness domain is, in part, a function of high psychosocial wellness scores prior to the start of the camp. Indeed, only two (of seven) participant pairs (4 of 14 individual participants) in our study had pre-camp scores below 3 ( = very good) in the DM management domain and the psychosocial wellness domain (Figure 1). Interestingly, both of these individuals used insulin pumps.

We observed a trend toward a perceived decrease in physical quality of life by both parents (*Δ—*5.36%) and campers (*Δ—*17.62%) from pre-camp to post-camp surveys. The reason(s) underlying this trend are at present unclear but may relate to several aspects, from the relatively low number of survey questions related to physical wellness (*n* = 4) to the daily agenda at Camp Sweeney, which may represent a significant increase in physical activity for many participants resulting in a certain level of physical discomfort, which is interpreted as a decrease in physical wellness. Previous studies at Camp Sweeney reported an average of *Δ*3% fat mass (−0.4 ± 0.1 kg weight) loss for participants [11], which may support the second hypothesis.

However, the significant finding of our study is the definitive and high degree of positive correlation between the degree of change in the diabetes management domain and the overall wellness, psychosocial wellness, and average quality of life (correlation coefficient = 0.9, 0.80, and 0.9, respectively, Table 3). A number of studies have investigated the effect of diabetes camps on parameters of mental health or an indicator of glycemic control. A limited number of studies, largely in adult patients, examined correlations between glycemic control and indexes of mental health. Self-efficacy, defined as confidence in one's ability to perform goal-directed behaviors when confronted with impediments, is a well-studied psychological construct consistently associated with better health outcomes. In patients with type 2 diabetes mellitus (T2DM), self-efficacy in the form of self-management played a central role in adequate glycemic control [16]. In another study in T2DM adult diabetic patients, a stronger correlation was observed between the severity of depression and acute symptoms of diabetes (hyper and hypoglycemia) than between glycemic control and diabetic complications [4]. Diabetes-related emotional distress and problems with self-management behaviors correlated with HbA1c in a short-term intensive treatment program in adults with T1DM [17]. A study showed that close to 40% of adult T2DM patients had alexithymia [18]. However, ours is the first study to directly address the impact of a diabetes camp on the correlation between diabetes management and psychosocial wellness.

Finally, while our results did not show significant differences between groups, our data analysis did show significant individual variation within groups, which we believe deserves further discussion. We observed two intriguing trends. First, greater improvement (*Δ* difference) was associated with lower absolute starting points in all domains except physical wellness (Figure 1). Second, there were significant differences between child and their parent's assessments across all domains; however, these differences are by far the greatest in the DM management domain (Figure 1). Obviously, the relatively low number of participants precludes any definitive conclusions being drawn from these observations.

## Figures and Tables

**Table 1:** Participant demographics.

Age (mean ± SD)	14.375 ± 2
Race/ethnicity	87.5% white, 12.5% mixed race
Age at DM diagnosis	7.375 ± 2.25
# Children in home (mean ± SD)	2.25 ± 0.75
# Caregivers in home	2
# Children with DM in home (mean ± SD)	1.375 ± 0.45
Caregivers with chronic illness (total in cohort)	2 Grave's disease, 2 adult onset essential hypertension

Abbreviation: DM, diabetes mellitus.

**Table 2:** The PedsQL survey was administered to attendees (children/campers) and their caregivers (parents/guardians) before the start of and after completion of Camp Sweeney.

Category	Child pre	Child post	Parent pre	Parent post	Child *Δ*	Parent *Δ*
DM management	3.07	3.25	3.21	3.38	17.86%	16.07%
Wellness	3.08	3.19	3.09	3.24	10.96%	14.54%
Physical	3.14	2.97	3.43	3.38	−17.62%	−5.36%
Psychosocial	3.07	3.24	3.04	3.22	16.25%	17.86%
Average QoL	3.09	3.16	3.19	3.30	6.86%	10.78%

*Note:* Likert scale scores of the PedsQL survey (0—never/none; 1—seldom/needs improvement; 2—sometimes/good; 3—often/very good; 4— all the time/excellent, respectively) were converted into numerical values (0 = 0; 1 = 0.25 [25%]; 2 = 0.50 [50%]; 3 = 0.75 [75%]; 4 = 1.00 [100%]). Numerical values were averaged for each of the four domains (DM management, overall wellness, physical wellness, and psychosocial wellness) to obtain the average quality of life (Average QoL). The differences between pre-camp and post-camp results were calculated separately for the campers (Child *Δ*) and their caregivers (Parent *Δ*) for all domains and analyzed by repeated, two-way ANOVA (no significant differences between groups were found).

Abbreviations: Average QoL, average quality of life (average of the four domains); DM, diabetes mellitus; PedsQL, standard Pediatric Quality of Life Inventory.

**Table 3:** Linear correlation analysis was performed on the difference of combined child and parent data (*Δ* Child and *Δ* Parent) for all domains.

Category	DM management	Wellness	Physical	Psychosocial	Average QoL
DM management	—	0.921*⁣*^*∗*^	0.446	0.798*⁣*^*∗*^	0.940*⁣*^*∗*^
Wellness	0.921*⁣*^*∗*^	—	0.459	0.929*⁣*^*∗*^	0.942*⁣*^*∗*^
Physical	0.446	0.459	—	0.231	−0.612
Psychosocial	0.798*⁣*^*∗*^	0.929*⁣*^*∗*^	0.231	—	0.752*⁣*^*∗*^
Average QoL	0.940*⁣*^*∗*^	0.942*⁣*^*∗*^	−0.612	0.752*⁣*^*∗*^	—

*Note:* The Pearson correlation coefficients (*R*^2^) for those correlations are reported, and highly significant positive correlations (*⁣*^*∗*^*p* < 0.05) are marked.

Abbreviations: Average QoL, average quality of life (average of the four domains); DM, diabetes mellitus.

## Data Availability

Data are openly available in a public repository that issues datasets with DOIs.

## References

[1] Diabetes and Quality of Life http://journal.diabetes.org/diabetesspectrum/00v13n1/pg21.htm.

[2] Bernstein C. M., Stockwell M. S., Gallagher M. P., Rosenthal S. L., Soren K. (2013). Mental Health Issues in Adolescents and Young Adults With Type 1 Diabetes: Prevalence and Impact on Glycemic Control. *Clinical Pediatrics*.

[3] Talakoub S., Nasiri M. (2012). Affective Responses of the Parents After Diagnosis of Type 1 Diabetes in Children. *Iranian Journal of Nursing and Midwifery Research*.

[4] Ludman E. J., Katon W., Russo J. (2004). Depression and Diabetes Symptom Burden. *General Hospital Psychiatry*.

[5] Statistics About Diabetes https://www.diabetes.org/resources/statistics/statistics-about-diabetes.

[6] Bultas M. W., Schmuke A. D., Moran V., Taylor J. (2016). Psychosocial Outcomes of Participating in Pediatric Diabetes Camp. *Public Health Nursing*.

[7] Santiprabhob J., Likitmaskul S., Kiattisakthavee P. (2008). Glycemic Control and the Psychosocial Benefits Gained by Patients With Type 1 Diabetes Mellitus Attending the Diabetes Camp. *Patient Education and Counseling*.

[8] Weissberg-Benchell J., Vesco A. T., Rychlik K. (2019). Diabetes Camp Still Matters: Relationships With Diabetes- Specific Distress, Strengths, and Self-Care Skills. *Pediatric Diabetes*.

[9] Gupta O. T., MacKenzie M., Burris A. (2018). White PC, Camp-Based Multi-Component Intervention for Families of Young Children With Type 1 Diabetes: A Pilot and Feasibility Study. *Pediatric Diabetes*.

[10] Hasan I., Chowdhury A. A., Haque M. I., Patterson C. C. (2021). Changes in Glycated Hemoglobin, Diabetes Knowledge, Quality of Life, and Anxiety in Children and Adolescents With Type 1 Diabetes Attending Summer Camps: A Systematic Review and Meta-Analysis. *Pediatric Diabetes*.

[11] Oden J. D., Franklin B., Fernandez E., Adhikari S., White P. C. (2018). Effects of Residential Summer Camp on Body Mass Index and Body Composition in Type 1 Diabetes. *Pediatric Diabetes*.

[12] The Sweeney Difference https://campsweeney.org/for-parents/why-camp-sweeney/.

[13] Varni J. W., Seid M., Rode C. A. (1999). The PedsQL™: Measurement Model for the Pediatric Quality of Life Inventory. *Medical Care*.

[14] Troncone A., Chianese A., Cascella C., Zanfardino A., Iafusco D. (2021). Psychological Outcomes in Children and Early Adolescents With Type 1 Diabetes Following Pediatric Diabetes Summer Camp: A 3-Month Follow-Up Study. *Frontiers in Pediatrics*.

[15] Ghazanfar H., Rizvi S. W., Khurram A., Orooj F., Qaiser I. (2016). Impact of Insulin Pump on Quality of Life of Diabetic Patients. *Indian Journal of Endocrinology and Metabolism*.

[16] O’Hea E. L., Moon S., Grothe K. B. (2009). The Interaction of Locus of Control, Self-Efficacy, and Outcome Expectancy in Relation to HbA1c in Medically Underserved Individuals With Type 2 Diabetes. *Journal of Behavioral Medicine*.

[17] Weinger K., Jacobson A. M. (2001). Psychosocial and Quality of Life Correlates of Glycemic Control During Intensive Treatment of Type 1 Diabetes. *Patient Education and Counseling*.

[18] Avci D., Kelleci M. (2016). Alexithymia in Patients With Type 2 Diabetes Mellitus: The Role of Anxiety, Depression, and Glycemic Control. *Patient Preference and Adherence*.

